# Occam’s razor gets a new edge: the use of symmetries in model selection

**DOI:** 10.1098/rsif.2022.0324

**Published:** 2022-08-24

**Authors:** Johannes G. Borgqvist, Sam Palmer

**Affiliations:** Wolfson Centre for Mathematical Biology, Mathematical Institute, University of Oxford, Oxford, UK

**Keywords:** symmetries, model selection, cancer, immunology, mechanistic modelling

## Abstract

We demonstrate the power of using symmetries for model selection in the context of mechanistic modelling. We analyse two different models called the *power law model* (PLM) and the *immunological model* (IM) describing the increase in cancer risk with age, due to mutation accumulation or immunosenescence, respectively. The IM fits several cancer types better than the PLM implying that it would be selected based on minimizing residuals. However, recently a symmetry-based method for model selection has been developed, which has been successfully used in an *in silico* setting to find the correct model when traditional model fitting has failed. Here, we apply this method in a real-world setting to investigate the mechanisms of carcinogenesis. First, we derive distinct symmetry transformations of the two models and then we select the model which not only fits the original data but is also invariant under transformations by its symmetry. Contrary to the initial conclusion, we conclude that the PLM realistically describes the mechanism underlying the colon cancer dataset. These conclusions agree with experimental knowledge, and this work demonstrates how a model selection criterion based on biological properties can be implemented using symmetries.

## 1. Introduction

Arguably one of the biggest challenges in mathematical biology is that of model selection. For numerous biological systems, the standard way of constructing models of a particular biological system is to propose a set of underlying mechanisms which are then translated into mathematical equations. Accordingly, each model consisting of a set of such equations encodes a number of biological properties and thus selecting one candidate model over another entails validating an underlying mechanism of the system of interest. Given numerous distinct candidate models describing some experimental data, the model selection problem is formulated as follows: *select the model that best fits the data*, and this is often referred to as *rejection based on residual analysis* [1]. However, as has been shown many contexts, e.g. modelling of cancer tumour growth [2], multiple distinct models can fit the same data equally well. To account for this problem, the initial model selection criterion is often modified based on the philosophical principle known as *Occam’s razor* initially formulated as *do not multiply entities beyond necessity* [3]. This implies that this modified model selection criterion becomes: *select the simplest model that best fits the data*. In the particular context of model selection based on residual analysis, the fit of a candidate model to experimental data is measured, while simultaneously penalizing the number of parameters, using, for example, the adjusted *R*^2^ value, the Akaike information criteria or the Bayes information criteria [1]. However, as these criteria are not based on biological properties of the studied system, there is no guarantee that the selected model is correct in the sense that it encodes the underlying mechanism, or that there does not exist some other, previously not considered, model that is in fact correct. To this end, we need an approach for selecting models that is based on fundamental biological principles, and here we can turn to mathematical physics for inspiration. Here, so-called *symmetries* have been used with huge success as they encode fundamental properties in terms of, for example, conservation laws such as energy conservation [4].

A symmetry is a transformation that leaves an object invariant. In the context of curves in general and differential equations in particular, a symmetry maps a (solution) curve to the same or another (solution) curve of the same type [5,6] meaning that symmetries preserve the structure of these curves. For example, power laws (functions of the form $f\;(x)=Cx^{\gamma }$) have scaling symmetries but not translation symmetries. Although symmetries are not frequently used in mathematical biology, they do play an important role in many biological systems [7] and in particular they have recently been successfully applied in a model selection scenario in the context of enzyme kinetics [8].

In this latter article by Ohlsson *et al.* [8], a single enzymatically catalysed reaction converting a substrate molecule to a product molecule was considered. The rate at which the enzyme consumes the substrate *S* is governed by the (dimensionless) Hill equation which can be formulated as follows [8]:

1.1 $$\frac{dS}{dt}=-\frac{S}{1+S^{n}},$$

and here the so-called *Hill coefficient*, *n*, corresponds to the number of substrate molecules required to produce one product molecule. In this context, an artificial model selection scenario was considered where three candidate models corresponding to the Hill coefficients *n* = 1, 2, 3 were fitted to simulated data using the same Hill coefficients. This implies that the correct model underlying the simulated data was known in advance; however, the traditional approach of residual analysis was unable to distinguish the three models. To employ the symmetry-based method, unique symmetries determined by the Hill coefficient of each candidate model were calculated. The simulated data were then transformed by each symmetry, then each model was fitted to transformed data generated by their respective symmetries and lastly, these fitted models were inversely transformed back so that their fit to the original data could be calculated (algorithm 1). Strikingly, the fit of the models with a *different Hill coefficient* from the one used to simulate the data was made worse as the data were transformed by their symmetries [8]. Better still, the fit of the correct model having the *same Hill coefficient* as the one that was used to simulate the data was unchanged no matter how much the data were transformed by the corresponding symmetry [8]. Consequently, when a known symmetry was built into the data, the fit of the correct model to transformed data was *invariant under transformations by its symmetry*.

In this article, we will implement the symmetry-based procedure for conducting model selection in a situation with actual experimental data. Specifically, the data consist of three time series of incidence rates of cancer in samples of patients as a function of their age in the case of the cancer types myeloma, colon cancer and chronic myeloid leukaemia (CML) [9,10]. Initially, we implement the standard model selection criteria based on the fit to this experimental data, and specifically we fit two of the models in [9] each corresponding to plausible biological hypotheses for the increase in cancer incidence due to ageing. Given the superior fit of one of these models to all three datasets, we select this model based on the standard model selection criteria. Then, we present a heuristic argument for the alternative and previously mentioned symmetry-based model selection criterion. Lastly, we implement this symmetry-based model selection criterion in [8] by deriving unique symmetries of our two candidate models which we then use in order to transform the data. In contrast to the standard model selection criteria based on the fit, we conclude based on the symmetry-based model selection criterion that the selected model realistically describes the myeloma and the CML datasets while the rejected model realistically describes the colon cancer dataset. Strikingly, these conclusions are supported by experimental evidence, indicating that symmetries in fact encode the biological mechanisms underlying the experimental data.

## 2. Results

### 2.1. A clear-cut case of model selection: one model has the best fit to the data of rate of cancer incidences as a function of age

There are two plausible hypotheses for the increased risk of developing cancer at a high age. The first one is an accumulation of genetic mutations due to ageing and the second one is a decline in the capacity of the immune system to clear mutated cells with age. These two biological mechanisms are the basis of the so-called *power law model* (*PLM*), originally from [11],

2.1 $$R(t)=At^{\gamma },$$

and the *immunological model* (*IM*),

2.2 $$R(t)=\frac{A}{\exp(e^{-\alpha (t-\tau )})-C},$$

from [9].

Here, *t* [years] is the age of the cancer patients and *R*(*t*) is the unitless incidence rate of cancer at age *t* corresponding to the risk of developing cancer. For the PLM in equation (2.1), the unitless parameter *γ* is related to the number of driver mutations required for cancer incidence and *A* [years^−*γ*^] is a scaling parameter. More specifically, *γ* is one less than the number of driver mutations where, for example, the value *γ* = 0 corresponds to one driver mutation being required to develop cancer and in that case the risk would be constant with respect to the age of the patients. For the IM in equation (2.2), the parameter *α* is the rate of decline of T-cell production, which is fixed to *α* = 0.044 yr^−1^ [9] for all cancer types, the parameter *τ* [years] is called the pivot age and *A* is a unitless scaling parameter. This model assumes that potentially cancerous cells can arise with equal probability at any age and that the number of cancer cells undergoes stochastic growth and can only progress to cancer incidence if a decreasing immune escape threshold (IET) is crossed. The parameter *C* is unitless and corresponds to shifting the IET by a fixed amount, such that values of *C* less than one correspond to a higher IET and more protection from cancer. For further details on both the PLM and the IM, see Material and methods.

Also, we note that equation (2.2) for the IM contains a double exponential for which we have chosen the notation ‘exp (e^*x*^)’. In practice, this means that the PLM has two parameters (*A*, *γ*) while the IM has three parameters (*A*, *τ*, *C*) that can be estimated by fitting these models to experimental data. The fitting of the models to the data is based on *orthogonal distance regression* (ODR) [12], and we use the summary statistic *root mean square* (RMS) to evaluate the fit of a model to the given data which is interpreted as follows: the lower the RMS value the better the fit (for more details on the model fitting, see Material and methods).

**Figure 1.**
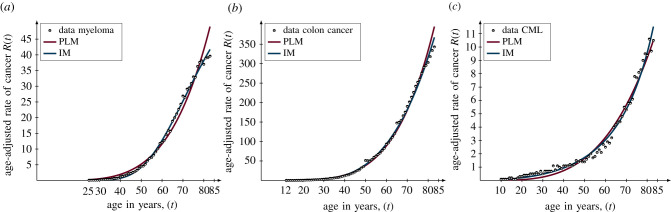
The fit of the candidate models to cancer data. The data in terms of the incidence rates of cancer as a function of age are illustrated by the black circles, the power law model (PLM) is illustrated by the dark magenta curves and the immunological model (IM) is illustrated by the dark blue curves. The fit is measured by the root mean square (RMS) based on orthogonal distance regression [12] in three cases. (*a*) *Myeloma*: RMS = 0.63 of the PLM and RMS = 0.36 of the IM. (*b*) *Colon cancer*: RMS = 0.62 of the PLM and RMS = 0.55 of the IM. (*c*) *Chronic myeloid leukaemia* (CML): RMS = 0.29 of the PLM and RMS = 0.21 of the IM.

When fitting the two candidate models to three time series corresponding to different cancer types, our newly adapted model IM is the better candidate model (figure 1 and table 1). In the case of myeloma, the IM has a fit of RMS = 0.36 compared to the PLM with a fit of RMS = 0.63. In the case of colon cancer, the IM has a fit of RMS = 0.55 compared to the PLM with a fit of RMS = 0.62. Lastly, in the case of CML, the IM has a fit of RMS = 0.21 versus the PLM with a fit of RMS = 0.29. Currently, the standard way of selecting a model is to choose the candidate model that best fits the data. Thus, based on this, we would select the IM as the appropriate candidate model, as well as concluding that an age-related decline in the immune system is the underlying mechanism for the increase in cancer incidences due to ageing. Also, since the IM has more parameters than the PLM, it is important to avoid overfitting to the data, and to resolve this problem, there are numerous statistical criteria for model selection that are ultimately based on *Occam’s razor*. Accordingly, the PLM might be favoured over the IM as it has fewer parameters which would imply that an accumulation of harmful mutations due to ageing is a more important factor for explaining the increase in incidences as a function of age. However, there are many ways of assessing goodness of fit which penalize for extra parameters in different ways and it is currently unclear which method to choose. Furthermore, the fits of both models are both so close that the differences are not even statistically significant for myeloma and CML using Vuong’s theory [13] (*p*-value > 0.05 for myeloma and CML and *p*-value = 0.001 for colon cancer).

**Table 1.** Fitting of the candidate models to the three datasets using orthogonal distance regression. The power law model (PLM) and the immunological model (IM) are fitted to three cancer datasets: myeloma, colon cancer and chronic myeloid leukaemia (CML). The fit is reported as the root mean square (RMS), and for the IM the parameter *α* is fixed to $\alpha =0.044\;{\mathrm{yr}}^{-1}$ [9].

	fit of models (RMS) and optimal parameters		
model	dataset		
	myeloma	colon cancer	CML
PLM	RMS = 0.63	RMS = 0.62	RMS = 0.29
	*A* = 1.53 × 10^−7^ ± 7.65 × 10^−8^	*A* = 5.78 × 10^−7^ ± 1.13 × 10^−7^	*A* = 1.12 × 10^−6^ ± 4.63 × 10^−7^
	*γ* = 4.43 ± 0.12	*γ* = 4.60 ± 0.047	*γ* = 3.63 ± 0.096
IM	RMS = 0.36	RMS = 0.55	RMS = 0.21
	*A* = 109.54 ± 11.47	*A* = 222.32 ± 13.18	*A* = 0.96 ± 0.29
	*τ* = 76.78 ± 0.89	*τ* = 64.10 ± 0.57	*τ* = 32.55 ± 5.60
	*C* = −0.49 ± 0.23	*C* = 0.94 ± 0.0028	*C* = 1.03 ± 0.0053

To this end, we need a better guiding principle for model selection which is based on the biological properties of the models at hand, which we propose can be achieved with a model selection framework based on *symmetries*.

### 2.2. A heuristic argument for the use of symmetries in model selection

When two or more models can fit data equally well, applying symmetries can provide extra information which ultimately can be used to distinguish between the models. This was demonstrated in the context of simulated data of enzymes described by the Hill function [8] and here the correct candidate model could be identified based on symmetries. We believe this may be the case in real-world data as well and to this end we present the following non-rigorous, heuristic argument.

Let *R*(*t*) be a model, e.g. the PLM in equation (2.1), and let $\Gamma_{\epsilon }$ be a transformation parameterized by a parameter *ε*. We say $\Gamma_{\epsilon }$ is a *symmetry* if it maps a solution curve (*t*, *R*(*t*)) to another solution curve $\left(\hat{t}(\epsilon ),\hat{R}(t,\epsilon )\right)$. In fact, we will restrict ourselves to symmetries that are $C^{\infty }$-diffeomorphisms, as these symmetries possess certain regularity properties. For a given model, the set *G* of such symmetry transformations together with a multiplication operation × constitute a *one-parameter Lie group of transformations*. This Lie group (*G*, × ) satisfies three conditions:

1. *Multiplication*: For two transformation parameters ϵ,δ∈R, multiplication of symmetries (meaning that we first transform with *δ* and then by *ε*) is defined by: $\Gamma_{\epsilon }\times \Gamma_{\delta }=\Gamma_{\epsilon +\delta }$.
2. *Identity element*: The trivial symmetry $\Gamma_{0}=\Gamma_{\epsilon =0}$ acts trivially on curves: $\Gamma_{0}(t,R(t))=(t,R(t))$.
3. *Inverse element*: The inverse symmetry is defined by $\Gamma_{\epsilon }^{-1}=\Gamma_{-\epsilon }$.

Given these properties of the Lie group (*G*, × ), it is straightforward to show that the following equation holds:

2.3 $$\Gamma_{-\epsilon }\times \Gamma_{\epsilon }(t,R(t))=(t,R(t)),$$

and this fundamental property is the basis for the symmetry-based framework for model selection. In particular, the interpretation of equation (2.3) is that if we initially transform a solution curve (*t*, *R*(*t*)) with a symmetry $\Gamma_{\epsilon }$ and then transform the transformed solution curve with $\Gamma_{-\epsilon }$, we come back to the original solution curve.

Moreover, suppose we have a time series with *m* data points denoted by $\left({\tilde{t}}_{i},{\tilde{R}}_{i}\right)$ for an index *i* = 1, …, *m*. Then, assuming an additive error model for simplicity, these data can be thought of as arising from a true model, *R*(*t*), along with small contributions, *e*_*i*_, for the response variable as well as *δ*_*i*_ for the explanatory variable, coming from either experimental noise or other mechanisms that contribute little to the dynamics:

2.4 $$\begin{array}{cc} {\tilde{R}}_{i}=R(t_{i})+e_{i}, & \left|e_{i}|<<|R(t_{i})\right|\\ {\tilde{t}}_{i}=t_{i}+\delta_{i}, & \left|\delta_{i}|<<|t_{i}\right| \end{array}\}\forall i=1,\ldots ,m.$$

Now, given the symmetry $\Gamma_{\epsilon }$, we can transform the data which we argue results in one of two different scenarios. In the first scenario, $\Gamma_{\epsilon }$ is a symmetry of the true underlying model, meaning that $\Gamma_{\epsilon }$ takes (*t*, *R*(*t*)) to another solution curve $\left(\hat{t}(\epsilon ),\hat{R}(t,\epsilon )\right)$. For small error terms, *e*_*i*_ and *δ*_*i*_, the transformed data will be fitted by a model close to the transformed model $\left(\hat{t}(\epsilon ),\hat{R}(t,\epsilon )\right)$, which will transform back to a model close to the original model under the inverse transform $\Gamma_{-\epsilon }$. In this scenario, the symmetry is manifest in the data.

In the second scenario, the symmetry is *not* manifest in the data which means that transformations by the symmetry will distort the time series. More precisely, transformations of the true underlying model (*t*, *R*(*t*)) by an inappropriate symmetry $\Gamma_{\epsilon }$ will result in another class of *systematic error terms*, in addition to those arising from *e*_*i*_ and *δ*_*i*_. Having two sources of potential error terms rather than one suggests that this will lead to a poor fit of the transformed data, which will then give a poor fit to the original data after the inverse transform $\Gamma_{-\epsilon }$ is applied.

Therefore, heuristically, if a dataset continues to be fitted well by a model after applying a transformation which is a symmetry of the model, then that can be considered evidence that the model is indeed underlying the dynamics. To the contrary, if systematic errors are introduced when the candidate model is fitted to data that are transformed by its symmetry, then the model does not underlie the dynamics. The steps of the symmetry-based procedure for model selection are summarized in algorithm 1. Here, we want to emphasize that this argument is in fact supported by a previous model selection scenario using simulated data where a symmetry of a known model was built in to the data, and in this case, the fit of the correct model to transformed data was invariant under transformation by its symmetry [8].


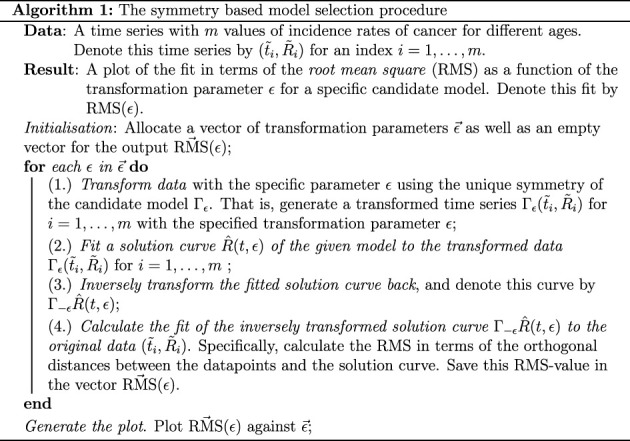


Importantly, each candidate model must have *unique* symmetries with respect to one another in order to be able to distinguish between the models, and thus we cannot implement a symmetry that is shared between candidate models. Provided this symmetry-based criterion for model selection, we will next present unique symmetries of our two candidate models.

### 2.3. Unique symmetries render the two candidate models distinguishable

To distinguish between the two candidates, we calculated two unique and comparable symmetries of each model. These symmetries are comparable in the sense that they are both unidirectional, or more precisely *t*-directional as they only transform the *t*-coordinate in any point (*t*, *R*(*t*)) they act on. Starting with the PLM, it has a *scaling symmetry* $\Gamma_{\epsilon }^{\mathrm{PLM}}$ given by

2.5 $$\Gamma_{\epsilon }^{\mathrm{PLM}}\;:\;(t,R(t))\mapsto (te^{\epsilon },R(t)).$$

To clarify the action of this symmetry, we can formulate an equation for any transformed curve obtained by this symmetry as follows:

2.6 $$\hat{R}(t,\epsilon )=A(\epsilon )t^{\gamma }\;\mathrm{and}\;A(\epsilon )=A\;e^{-\gamma \epsilon }.$$

Here, it is clear that this symmetry transforms solution curves by altering the scaling parameter *A* of the PLM in equation (2.1), and thus the symmetry preserves the number of driver mutations for a specific cancer type given by *γ*. Similarly, the IM has a symmetry $\Gamma_{\epsilon }^{\mathrm{IM}}$ given by

2.7 $$\Gamma_{\epsilon }^{\mathrm{IM}}\;:\;(t,R(t))\mapsto \left(\tau -\frac{\ln(\ln(\exp(e^{-\alpha (t-\tau )})-\alpha \;e^{\alpha \tau }\epsilon ))}{\alpha },R(t)\right).$$

Although the symmetry $\Gamma_{\epsilon }^{\mathrm{IM}}$ looks complicated at first, its action also corresponds to perturbations of a single parameter which is clear by formulating the corresponding equation for any transformed curve obtained by this symmetry given by

2.8 $$\hat{R}(t,\epsilon )=\frac{A}{\exp(e^{-\alpha (t-\tau )})-C(\epsilon )},\;C(\epsilon )=C-\alpha \;e^{\alpha \tau }\epsilon .$$

**Figure 2.**
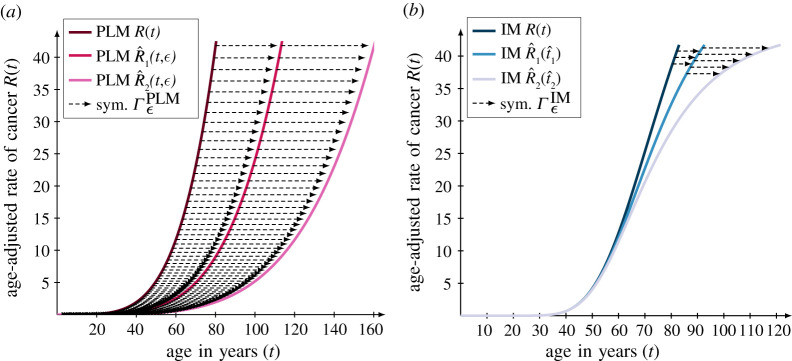
Unique symmetries render the two candidate models distinguishable. The actions of the *t*-directional symmetries of the two candidate models are illustrated when the symmetries transform a solution curve twice with a fixed transformation parameter *ε*. The two symmetries are: (*a*) the scaling symmetry $\Gamma_{\epsilon }^{\;\mathrm{PLM}}$ of the PLM where the original solution is defined by the parameters (*A*, *γ*) = (1.53 × 10^−7^, 4.43) with a transformation parameter of $\epsilon =\epsilon_{\mathrm{scale}}^{\mathrm{PLM}}/2=\ln(2)/2\approx 0.35$ in accordance with equation (2.11) and (*b*) the symmetry $\Gamma_{\epsilon }^{\;\mathrm{IM}}$ of the IM where the original solution is defined by the parameters (*A*, *τ*, *C*, *α*) = (109.54, 76.78, − 0.49, 0.044) with a transformation parameter of $\epsilon =\epsilon_{\mathrm{scale}}^{\;\mathrm{IM}}/2\approx 0.38$ in accordance with equation (2.12).

Here, it is the parameter *C* of the IM in equation (2.2) that is altered by the symmetry $\Gamma_{\epsilon }^{\;\mathrm{IM}}$. Also, the symmetry $\Gamma_{\epsilon }^{\;\mathrm{IM}}$ preserves the decline rate of the immune system *α* as well as the pivot age *τ* of the specific cancer type. Consequently, transformations by the symmetries of the respective models, i.e. $\Gamma_{\epsilon }^{\;\mathrm{PLM}}$ and $\Gamma_{\epsilon }^{\;\mathrm{IM}}$, produce remarkably different solution curves (figure 2) and this fact enables us to distinguish between these two seemingly similar candidate models. Another, aspect that distinguishes the two symmetries is that $\Gamma_{\epsilon }^{\mathrm{PLM}}$ is *parameter independent* in the sense that it is independent of the parameters (*A*, *γ*) of the PLM in equation (2.1) while $\Gamma_{\epsilon }^{\;\mathrm{IM}}$ is *parameter dependent* as it depends on the parameters (*α*, *τ*) of the IM in equation (2.2). This difference affects the scale of the transformation parameter *ε* determining the extent to which the curves and the data are transformed.

To be able to compare the effect of the symmetries of our candidate models, we introduce the notion of a *transformation scale* denoted by *ε*_scale_ for our two models and three datasets. Since both symmetries are *t*-directional, they move the original *t*-coordinate of *t* years to a transformed *t*-coordinate of $\hat{t}(\epsilon )$ years where $\hat{t}(\epsilon )>t$ for *ε* > 0. Thus, if we want to transform the data so that a specific age of *t* years is, say, doubled, we want to find the transformation parameter *ε*_scale_ so that $\hat{t}(\epsilon_{\mathrm{scale}})=nt$ for a factor *n* = 2. To this end, we ask ourselves the following question: which transformation parameters *ε*_scale_ of the two models will move the data point (*t*, *R*(*t*)) to the data point (*nt*, *R*(*t*)) for a given age of *t* years and some factor *n* > 1? In the case of the PLM, this transformation scale is a simple function of the factor *n* given by

2.9 $$\epsilon_{\mathrm{scale}}^{\mathrm{PLM}}=\ln(n).$$

In the case of the IM, this transformation scale is given by the following equation:

2.10 $$\epsilon_{\mathrm{scale}}^{\;\mathrm{IM}}=\frac{\exp(e^{-\alpha (t-\tau )})-\exp(e^{-\alpha (nt-\tau )})}{\alpha e^{\alpha \tau }},$$

and hence this scale is dependent on the parameters (*α*, *τ*) and the age *t* *in addition* to the factor *n*. To get a grasp of the order of magnitude of these scales, consider the transformation scales corresponding to a doubling of the maximum age of *t* = 85 years in the time series, i.e. so that $\hat{t}(\epsilon_{\mathrm{scale}})=170\;\mathrm{years}$. In particular, these transformation scales are calculated by plugging in the values (*t*, *n*) = (85, 2) in equation (2.9) and equation (2.10) respectively. In the case of the PLM, the transformation scale for all three datasets is given by

2.11 $$\epsilon_{\mathrm{scale}}^{\mathrm{PLM}}=\ln(2)\approx 0.69,$$

according to equation (2.9). In the case of the IM, the transformation scales for the three datasets are obtained by plugging in the optimal values for the parameter *τ* in table 1, the value *α* = 0.044 yr^−1^ in addition to the values (*t*, *n*) = (85, 2) in equation (2.10). These resulting transformation scales are the following:

2.12 $$\begin{array}{rl} & \mathrm{myeloma}\;:\;\;\epsilon_{\mathrm{scale}}^{\;\mathrm{IM}}=0.77,\\ & \mathrm{colon}\;\mathrm{cancer}\;:\;\;\epsilon_{\mathrm{scale}}^{\;\mathrm{IM}}=0.65\\ \mathrm{and}\;\; & \mathrm{CML}\;:\;\;\;\;\epsilon_{\mathrm{scale}}^{\;\mathrm{IM}}=0.55. \end{array}\}$$

In summary, the transformation scale for the PLM is independent of the parameters of the model while the transformation scale of the IM is dependent on the parameters *α* and *τ* of the model. All details behind the calculations of these symmetries as well as the derivation of their properties can be found in the electronic supplementary material. Provided these unique symmetries of our two candidate models, we will next implement them in the symmetry-based model selection procedure in algorithm 1.

### 2.4. The symmetry-based methodology reveals the biological mechanism underlying the data

**Figure 3.**
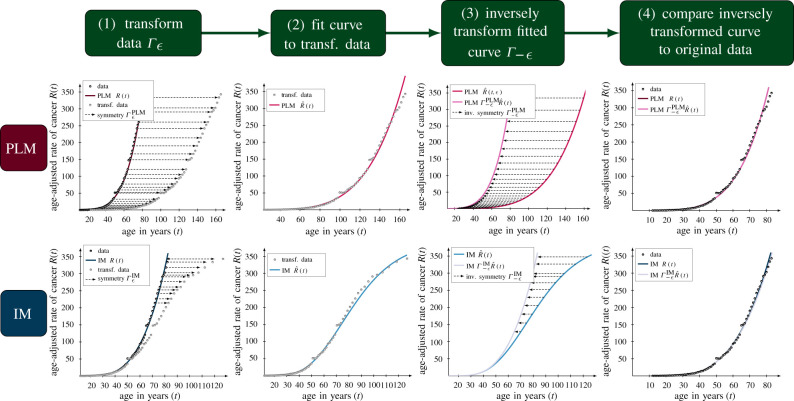
The detailed steps of the symmetry-based framework for model selection. The four steps of the symmetry-based framework for model selections are illustrated for the PLM in the top row and the IM in the bottom row. In all cases, the symmetry-based framework is implemented with the optimal parameters of both models obtained from the initial model fitting to the colon cancer data (table 1). The framework is illustrated for the transformation parameters $\epsilon =\epsilon_{\mathrm{scale}}^{\mathrm{PLM}}=\ln(2)\approx 0.69$ in the case of the PLM in the top row in accordance with equation (2.11) and $\epsilon =\epsilon_{\mathrm{scale}}^{\mathrm{IM}-\mathrm{III}}\approx 0.65$ in the case of the IM in the bottom row in accordance with equation (2.12).

Starting from the optimal parameters obtained from the initial model fitting (table 1), we implemented the symmetry-based framework for model selection (figure 3). Here, the criterion for model selection is the following: *select a model which fits the original data and whose fit to transformed data is invariant under transformations by its symmetries*. Based on this, we analysed the fit of the candidate models to transformed data using algorithm 1.

**Figure 4.**
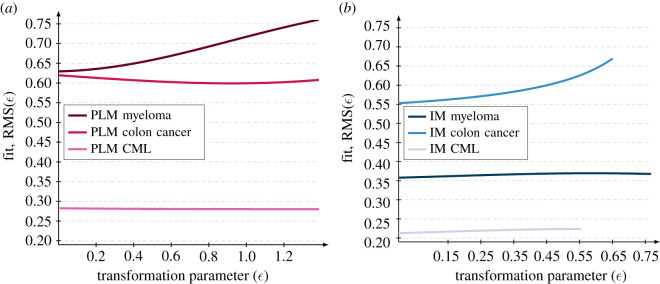
The symmetry-based framework for model selection reveals the underlying mechanisms for each cancer type. The fit RMS(*ε*) to transformed data is plotted against the transformation parameter *ε* in two cases. (*a*) The PLM fitted to all three datasets on a transformation scale of $\epsilon \in [0,2\;\epsilon_{\mathrm{scale}}^{\mathrm{PLM}}]$ where the upper bound is given by equation (2.11). (*b*) The IM fitted to all three datasets on three different transformation scales given by $\epsilon \in [0,\epsilon_{\mathrm{scale}}^{\;\mathrm{IM}}]$ where the upper bounds are given by equation (2.12). In all cases, the symmetry-based framework is implemented with the optimal parameters of both models obtained from the initial model fitting (table 1). Based on the criteria stating that a model is selected if it fits the original data and if its fit to transformed data is invariant under transformations by its symmetry, the IM is a realistic model of the myeloma and CML datasets while the PLM is a realistic model of the colon cancer dataset.

We find that all datasets are preserved by the model symmetries except for two: the myeloma dataset under the PLM symmetry (figure 4*a*) and the colon cancer dataset under the IM symmetry (figure 4*b*). From this, we conclude that the symmetry-based framework favours the PLM for colon cancer, the IM for myeloma and does not distinguish between models for CML. We would then update our initial findings that the IM fits better than the PLM for each cancer type, to instead conclude that the PLM explains colon cancer while the IM explains myeloma and CML. Biologically, this would suggest that the primary mechanism behind the rise in cancer risk with age is mutation accumulation for colon cancer and immune system decline for myeloma and CML.

Strikingly, these conclusions tie in with what is already known about the mechanisms of carcinogenesis for these cancer types. People with germline mutations in POLE/POLD or DNA mismatch repair genes accumulate mutations at a faster rate, which would disproportionately increase cancer risk in cancer types with a higher number of driver mutations. In fact, this only leads to a small increase in cancer risk, except for colorectal and endometrial cancers, suggesting that those are the cancer types for which mutation accumulation plays the biggest role [14]. Furthermore, analysis of shared mutations in tumour samples has been used to estimate the number of driver mutations in each cancer type and once again colorectal and endometrial cancers are the top two cancer types [15]. The ratio of risk of colon cancer, in those without and with an inherited driver mutation in the gene APC, rises linearly with age [16], supporting the PLM for colon cancer. On the other extreme, CML is characterized by just one driver mutation, the mitogenic fusion protein BCR-ABL, also referred to as the Philadelphia chromosome [15,17]. With just one driver mutation, there would be no increase in risk with age due to mutation accumulation and the increase in risk would be entirely explained by immune system decline. In fact, CML is very well fitted by just a pure exponential, with risk doubling every 16 years [9]. This makes CML risk inversely proportional to T-cell production, suggesting that immune system decline is indeed the only factor behind the increase in risk with age. Furthermore, several infectious diseases including COVID-19 [18] also double in risk for every 16 years of age, possibly indicating a shared mechanism of disease progression due to immune system decline [9].

## 3. Discussion

In this work, we have implemented a symmetry-based criterion for model selection using experimental data in the context of the effect of ageing on cancer incidence. Given three time series of the rate of incidences of myeloma, colon cancer and CML in samples of patients in the age span 0–85 years, as well as two plausible mechanistic models called the PLM and the IM, we select the latter of these models based on the standard model selection criteria as it fits the data better than the former model. Then, we present a heuristic argument for using symmetries in model selection, where a model should be selected if it can fit data that are transformed by its symmetries as this implies that the symmetries of the model are also manifest in the data. Thereafter, we derive two unique symmetries of the PLM and the IM, which are given by $\Gamma_{\epsilon }^{\mathrm{PLM}}$ in equation (2.5) and $\Gamma_{\epsilon }^{\;\mathrm{IM}}$ in equation (2.7) respectively, which, in turn, renders the two models distinguishable. Lastly, we implement these symmetries in the symmetry-based procedure for model selection where the following four steps are repeated for multiple transformation parameters *ε*. Firstly, the data are transformed by the symmetries of the candidate models, secondly, the candidate models are fitted to the transformed data, thirdly, these fitted models are inversely transformed back and fourthly, the fit of inversely transformed models to the original data is calculated. These steps are described in detail in algorithm 1 and shown graphically in figure 3.

In the case of the IM, the fits to the transformed myeloma and CML datasets are invariant and similarly the fits of the PLM to the transformed colon cancer and CML datasets are invariant. Provided these results, we update our initial conclusions and select the IM for myeloma as well as CML risk and the PLM for colon cancer risk. These conclusions are supported by experimental evidence where colon cancer is thought to be one of the top two cancer types (along with endometrial cancer) for which mutation accumulation plays the biggest role (highest number of driver mutations). On the other extreme, CML is characterized by just one driver mutation and risk rises with age almost exactly inversely proportional to T-cell production. This risk behaviour is shared with several infectious diseases, suggesting a similar, immunological mechanism for the increase in risk with age.

An advantage with the symmetry-based model selection procedure is that it can indicate if all candidate models in the context of model selection are incorrect. Under the classical model selection criterion that is based on the fits of the candidate models, an implicit assumption of the modeller is that one model is correct, i.e. the one with the best fit, whereas the other candidates are incorrect. However, as most models are constructed using numerous assumptions which modellers are forced to make due to, for example, a lack of knowledge about the studied system, it is highly plausible that *all candidate models* are incorrect. Thus, by framing it as a model selection problem forcing the modeller to pick one of the candidate models, an incorrect model might be selected resulting in a poor capacity to extrapolate from the observed data as well as a poor predictive capacity of the selected model. On the other hand, the symmetry-based model selection criterion can reveal if none of the candidate models capture the mechanism underlying the data. In particular, this would correspond to a situation where the RMS of all candidate models are increasing functions of the transformation parameter *ε*, such as the RMS of the PLM fitted to the myeloma dataset (figure 4*a*) and the RMS of the IM fitted to the colon cancer dataset (figure 4*b*). If all models are rejected this provides important negative information indicating that the modeller should construct new candidate models. In addition, as demonstrated in this work in the case of the colon cancer data, we would select an incorrect model, namely the IM, based on the fit to the original data, while the symmetries of the candidate model in fact reveal that the rejected model, i.e. the PLM, in fact captures the underlying mechanism. Moreover, in this work, we have implemented the symmetry-based framework for two minimal models, and it is only reasonable to ask whether or not the same methodology can be implemented on larger models.

The difficulty of finding the symmetries of the candidate models increases with the number of explanatory and response variables. In the case of the most general model formulated as a coupled system of nonlinear PDEs with *x* explanatory variables and *p* response variables, the corresponding so-called *linearized symmetry conditions*, equation (4.16), that must be solved to find the symmetries constitute another coupled system of nonlinear PDEs of *p* equations in *p* + *x* variables. In this work, where we have one explanatory variable *t* and one response variable *R*(*t*), we can calculate the symmetries by hand as we need to solve a single nonlinear PDE in two variables, but this is of course not possible to do in the general case. Therefore, it is not surprising that the symmetry-based analysis thus far is restricted to low-dimensional models, which is a potential problem in mathematical biology as numerous models are high dimensional. One potential solution to this problem is to implement automated and computer assisted algorithms for solving the linearized symmetry conditions stemming from high-dimensional models [19,20]. On the other hand, an arguably more interesting approach to this problem that symmetries offer is to *re-formulate the problem from a model selection problem to a model construction problem*. By definition, a model selection problem starts from a set of candidate models, where the number of candidate models is restricted by the knowledge about the studied system as well as the imagination of the modeller, and given this starting point the modeller is forced to calculate the symmetries of these potentially high-dimensional models. On the other hand, symmetries allow modellers to *construct models starting from the symmetries* by using the so-called *differential invariants* of these symmetries [19]. Thus, if we can interpret biological properties in terms of a set of symmetries, we can build-in these properties into the very structure of the constructed models by constructing the models from the differential invariants of the symmetries.

Given the broad range of applications for symmetry methods, we believe that they constitute a powerful tool in mathematical biology. Currently, these methods have been used as a tool for performing identifiability analysis [20–23] in the context of biological data and first-order ODEs, and other recent works demonstrate that symmetries play a role in model construction [19]. Moreover, symmetries recently played a pivotal role in a remarkable result obtained from deep-learning models, where spatial symmetries were incorporated in the input data as statistical priors which resulted in the discovery of protein structures beginning with only an amino acid sequence [24,25]. Hence, researchers in mathematical modelling of biological systems are beginning to take advantage of these powerful symmetry methods, and this work constitutes another stepping stone for elucidating mechanisms of biological systems using symmetries.

## 4. Material and methods

All the details about the mathematical theory of symmetry methods, the derivation of the symmetries as well as the validation of these symmetries can be found in the electronic supplementary material.

### 4.1. The candidate models

The PLM for carcinogenesis was formulated by Armitage & Doll [11] and is based on the concept of cancer risk rising with age due to accumulating genetic mutations. Assuming independent Poisson processes occurring at the same rate, the probability of accumulating *n* mutations in a cell by age *t* is of the form (1 − e^−*λt*^)^*n*^, for some rate *λ*. The risk of developing cancer at age *t* is then given by the derivative and, after taking a rare-event (small *λ*) approximation, we arrive at the PLM

4.1 $$R(t)=At^{\gamma },$$

where the unitless parameter *γ* = *n* − 1 and *A* is a scaling parameter given by *A* = *nλ*^*n*^.

Another plausible mechanism for the increase in cancer risk with age is that the ability to control nascent neoantigens is impaired as the immune system declines. In particular, cancerous neoantigens are recognized by T-cells, which develop in the thymus. The volume of the thymus and the production of T-cell clones decrease exponentially with age, halving every 16 years, starting from puberty. The IM of [9] assumes that potentially cancerous cells can originate with equal probability at any age, at a rate *r*, and that the number of such cells undergoes a random walk, with a birth rate *b* and a death rate *d*. The absorbing states are at zero cells, corresponding to tumour eradication, and *K* cells, where *K* is an IET. The IET is further assumed to decrease exponentially at the same rate as the decrease in T-cell production. That is,

4.2 $$K=K_{0}\;e^{-\alpha t}+K_{1},\;\alpha =0.044\;{\mathrm{yr}}^{-1},$$

for some constants *K*_0_ and *K*_1_.

Under stochastic growth, the probability of reaching the IET, *K*, is given by

4.3 $$b^{K-1}\frac{d-b}{d^{K}-b^{K}}.$$

Putting this together gives the risk at age *t* as

4.4 $$R(t)=\frac{A_{0}}{\exp(B_{0}\;e^{-\alpha t}+B_{1})-1},$$

where *A*_0_ = *r*(*d* − *b*)/*b*, *B*_0_ = *K*_0_ln(*d*/*b*) and *B*_1_ = *K*_1_ln(*d*/*b*). Finally, restricting to the parameter space where *d* > *b*, as is the case when fitting to our three cancer datasets, and defining *τ* = ln(*B*_0_)/*α*, we arrive at the IM

4.5 $$R(t)=\frac{A}{\exp(B\;e^{-\alpha (t-\tau )})-C},$$

where *A* = *A*_0_exp(−*K*_1_ln(*d*/*b*)) and *C* = exp( − *K*_1_ln(*d*/*b*)). When *K*_1_ > 0, corresponding to a higher IET, we get values of *C* < 1 and a reduction in cancer risk, especially at late ages.

The IM above contains two sub-models. Setting *C* = 1 gives a two-parameter model,

4.6 $$R(t)=\frac{A}{\exp(e^{-\alpha (t-\tau )})-1},$$

and further restricting to *d* = *b* (for an unbiased random walk) gives a one-parameter model,

4.7 $$R(t)=A\;e^{\alpha t}.$$

These are referred to, in [9], as IM-II and IM-I, respectively. In fact, these are the only models in [9] and we have generalized to *C* ≠ 1 because the method for deriving symmetries treats the IM and IM-II the same and the resulting symmetries are symmetries of the more general IM. See electronic supplementary material for the full derivation.

The symmetry $\Gamma_{\epsilon }^{\;\mathrm{IM}}$ used in this article is a symmetry of the IM, but not IM-I or IM-II. Similarly, there is a symmetry of the IM and IM-II, but not IM-I, given by

4.8 $$\Gamma_{\epsilon }^{\mathrm{IM}-\mathrm{II}}\;:\;(t,R(t))\mapsto \left(\tau -\frac{\ln(\ln(\exp(e^{-\alpha (t-\tau )}+\epsilon )+1-e^{\epsilon }))}{\alpha },R(t)\right),$$

and a symmetry of all three models, IM, IM-II and IM-I, given by

4.9 $$\Gamma_{\epsilon }^{\mathrm{IM}-I}\;:\;(t,R(t))\mapsto (t+\epsilon ,R(t)).$$

This latter symmetry is just a time translation symmetry, which is apparent from the appearance of *t* in exponentials in each model.

For all three cancer datasets, applying algorithm 1 using either the symmetry $\Gamma^{\;\mathrm{IM}-\mathrm{II}}$ or $\Gamma^{\;\mathrm{IM}-I}$ results in good fitting for all *ε* up to *ε*_scale_ (data not shown). Therefore, these symmetries are manifest in the data for all three cancer types and our overall conclusions are not affected by considering these additional symmetries.

### 4.2. Calculating the symmetries of each model

A symmetry is an operator which maps a solution curve of an *ordinary differential equation* (ODE) to another solution curve [6]. Let *γ* = (*t*, *R*(*t*)) be a solution curve to the single first-order ODE given by

4.10 $$\frac{dR}{dt}=\omega (t,R),$$

where the function *ω* corresponds to the reaction term. Then a (point-wise) *symmetry* of this ODE is an operator of the type

$$\Gamma_{\epsilon }\;:\;(t,R)\mapsto (\hat{t}(\epsilon ),\hat{R}(\epsilon )),$$

which maps a solution curve *γ* = (*t*, *R*(*t*)) to another solution curve $\hat{\gamma }=(\hat{t}(\epsilon ),\hat{R}(\epsilon ))$. A restriction of this work is to focus on so-called $C^{\infty }$-diffeomorphisms parameterized by a single *transformation parameter* *ε* which implies that the target functions $\hat{t}(\epsilon )$ and $\hat{R}(\epsilon )$ are continuous functions of *ε*. Using this latter fact, it is possible to write the target functions as Taylor expansions locally around *ε* ≈ 0 as follows:

4.11 $$\hat{t}(\epsilon )=t+\xi (t,R)\epsilon +O(\epsilon^{2})$$

and

4.12 $$\hat{R}(\epsilon )=R+\eta (t,R)\epsilon +O(\epsilon^{2}).$$

The so-called tangents *ξ* and *η* define the following vector field:

4.13 $$X=\xi (t,R)\partial_{t}+\eta (t,R)\partial_{R},$$

which is referred to as the *infinitesimal generator of the Lie group* [6]. Using this local description of the action of the symmetry $\Gamma_{\epsilon }$, it is possible to retrieve the global behaviour through the *exponential map* which is defined as follows:

4.14 $$e^{\epsilon X}=\sum_{\;j=0}^{\infty }\frac{\epsilon^{j}}{j!}X^{j}.$$

More precisely, it is possible to generate a symmetry $\Gamma_{\epsilon }$ using its infinitesimal generator *X* according to the following equation:

4.15 $$\Gamma_{\epsilon }\;:\;(t,R)\mapsto (e^{\epsilon X}t,e^{\epsilon X}R).$$

Thus, it is sufficient to calculate the infinitesimal generator *X* since the corresponding symmetry $\Gamma_{\epsilon }$ is obtained by the exponential map according to the above equation. The tangents *ξ* and *η* in the infinitesimal generator of the Lie group *X* are found by solving the so-called *linearized symmetry condition* [6] defined as follows:

4.16 $$\frac{\partial \eta }{\partial t}+\left(\frac{\partial \eta }{\partial R}-\frac{\partial \xi }{\partial t}\right)\omega (t,R)-\frac{\partial \xi }{\partial R}\omega {\left(t,R\right)}^{2}=\xi \frac{\partial \omega }{\partial t}+\eta \frac{\partial \omega }{\partial R}.$$

Furthermore, a symmetry can be characterized as either *trivial* or *non-trivial* by using the *reduced characteristic* [6] denoted by $\bar{Q}$. For a first-order ODE, it is defined as follows:

4.17 $$\bar{Q}(X)=\bar{Q}(t,R){|}_{\xi ,\eta \;\mathrm{defined}\;\mathrm{by}\;X}=\eta (t,R)-\omega (t,R)\xi (t,R).$$

If $\bar{Q}(X)\equiv 0$ then the symmetry is *trivial* implying that it does not move any data points otherwise the symmetry is *non-trivial*. In the symmetry-based methodology for model selection, only non-trivial symmetries are implemented.

Since the candidate models are formulated as curves, their symmetries are found by firstly re-formulating these curves as ODEs and secondly the linearized symmetry condition is solved using the ODE in each case (for all the details involving the calculations of the symmetries as well as their validation, see the electronic supplementary material).

### 4.3. Fitting of the candidate models

The time-series data of the increase in incidences of cancer due to ageing have been collected from [9,10]. Specifically, we focused on three time series based on a sample of patients in the age span from zero to 85 years, and these corresponded to three different cancer types: myeloma, colon cancer and CML. Moreover, we excluded data points corresponding to zero incidences of cancer implying that we removed datapoints corresponding to young patients. More precisely, in the case of myeloma, we removed patients under the age of 25 years, in the case of the colon cancer patients, we removed patients under the age of 12 years and in the case of CML, we removed patients under the age of 10 years. As we described previously, we assumed that there are noise terms contributing to random errors in both the incidence rate of cancer *and* the biological age of the patients, so next we will describe the underlying statistical assumption of the model fitting.

To evaluate the fit of the transformed data to a transformed solution curve, we use ODR which is encoded in the RMS value [12]. For the sake of notation, assume that we have *m* data points $\left({\tilde{t}}_{i},{\tilde{R}}_{i}\right)$ for an index *i* = 1, …, *m* in a time series. Moreover, assume that we have an equation for any transformed solution curve of the model of interest such as equation (2.6) of the PLM or equation (2.8) of the IM, and denote this transformed solution curve by $\hat{R}(t,\epsilon )$. Then, the RMS value as a function of the transformation parameter *ε* is defined as follows:

4.18 $$\mathrm{RMS}(\epsilon )=\sqrt{\frac{\mathrm{SS}(\epsilon )}{m}},\;\mathrm{SS}(\epsilon )=\sum_{i=1}^{m}{\left({\tilde{t}}_{i}-t_{i}\right)}^{2}+{\left({\tilde{R}}_{i}-\Gamma_{-\epsilon }\hat{R}(t_{i},\epsilon )\right)}^{2}.$$

In equation 4.18), the coordinates *t*_*i*_ for *i* = 1, …, *m* are chosen so that the distances between the data points $\left({\tilde{t}}_{i},{\tilde{R}}_{i}\right)$ and the points on the inversely transformed curves $\left(t_{i},\Gamma_{-\epsilon }\hat{R}(t_{i},\epsilon )\right)$ are minimized. In fact, we can write down an explicit expression for these coordinates, as they are chosen based on orthogonality. More precisely, the vector

4.19 $$v_{1}=\left(\begin{array}{c} \Gamma_{\epsilon }{\tilde{t}}_{i}-t_{i}\\ \Gamma_{\epsilon }{\tilde{R}}_{i}-\Gamma_{-\epsilon }\hat{R}(t_{i},\epsilon ) \end{array}\right)\;$$

should be orthogonal to the tangent vector at the point (*t*_*i*_, *R*(*t*_*i*_)) given by

4.20 $$v_{2}=\left(\begin{array}{c} 1\\ {\frac{dR}{dt}|}_{t=t_{i}} \end{array}\right).$$

By using the standard Euclidean dot product between two vectors in $R^{2}$ as follows:

4.21 $$\begin{array}{rl} & \langle v_{1},v_{2}\rangle =v_{1}^{T}v_{2}=\left(\begin{array}{cc} {\tilde{t}}_{i}-t_{i} & {\tilde{R}}_{i}-\Gamma_{-\epsilon }\hat{R}(t_{i},\epsilon ) \end{array}\right)\left(\begin{array}{c} 1\\ {\frac{dR}{dt}|}_{t=t_{i}} \end{array}\right)\\ & =({\tilde{t}}_{i}-t_{i})+{\frac{dR}{dt}|}_{t=t_{i}}({\tilde{R}}_{i}-\Gamma_{-\epsilon }\hat{R}(t_{i},\epsilon )) \end{array}$$

we have that the two vectors are orthogonal if 〈***v***_1_, ***v***_2_〉 = 0. Hence, we choose the coordinates *t*_*i*_ on the transformed curves $\hat{R}(t,\epsilon )$ so that they solve the following equations:

4.22 $$({\tilde{t}}_{i}-t_{i})+{\frac{dR}{dt}|}_{t=t_{i}}({\tilde{R}}_{i}-\Gamma_{-\epsilon }\hat{R}(t_{i},\epsilon ))=0,\;i=1,\ldots ,m.$$

In our implementation, we do not solve equation (4.22) but instead we use the built-in function *fmin_cobyla* in *SciPy* [28] to find the points $\left(t_{i},\Gamma_{-\epsilon }\hat{R}(t_{i},\epsilon )\right)$ on the inversely transformed solution curves, and this function conducts a local minimization in the sense that it finds the local minimum that is closest to a provided start guess in terms of the points $\left(t_{i},\Gamma_{-\epsilon }\hat{R}(t_{i},\epsilon )\right)$.

Also, we use equation (4.18) to calculate the RMS value of the data points in the original times series (*t*_*i*_, *R*_*i*_) and the orthogonal points on the original solution curves *R*(*t*), i.e. equation (2.1) for the PLM and equation (2.2) for the IM. This RMS value is calculated by plugging in *ε* = 0 in equation (4.18) or, in other words, this value corresponds to RMS(0) in equation (4.18).

Moreover, in step 2 of algorithm 1 when the two models are fitted to the transformed data, we only fit a single parameter in both cases. In the case of the PLM, it is only the parameter *A* that it is fitted to the transformed data since this is the only parameter that the symmetry $\Gamma_{\epsilon }^{\mathrm{PLM}}$ alters according equation (2.6). In this step, we used a single start guess for the parameter *A* given by

4.23 $$\tilde{A}\;e^{-\tilde{\gamma }\epsilon },$$

where $\left(\tilde{A},\tilde{\gamma }\right)$ correspond to the optimal parameters obtained by fitting the PLM to the original data and *ε* is the transformation parameter with which the original data were transformed. Also, we kept the parameter *γ* fixed to the optimal value obtained by fitting the PLM to the original time series. Similarly, in the case of the IM, it is only the parameter *C* that is fitted to the transformed data since this is the only parameter that the symmetry $\Gamma_{\epsilon }^{\mathrm{IM}-\mathrm{III}}$ alters according to equation (2.8). In this step, we used 10 linearly spaced start guesses in the following interval:

4.24 C∈[−5,1].

Again, we kept the parameters (*A*, *τ*) fixed to the optimal values obtained by fitting the IM to the original time series, and the parameter *α* was fixed to the value $\alpha =0.044\;{\mathrm{yr}}^{-1}$ [9].

Furthermore, in step 3 of algorithm 1 when the fitted models are inversely transformed using $\Gamma_{\epsilon }^{-1}$, the explicit equations for the transformed solution curves are used. More precisely, to obtain the inversely transformed solution curve of the PLM, the parameter *A* is calculated using the equation for *A*(−*ε*) in equation (2.6). Similarly, the inversely transformed solution curve of the IM is given by the equation for *C*(−*ε*) in equation (2.8).

Also, we conducted the Vuong test for non-nested models [29] in R [30] using the packages drc [31] and nonnest2 [32]. This test was implemented to see whether there was a significant difference in terms of the fit of the two models to the three datasets. The implementation of this script can be accessed at the GitHub repository associated with this article [26].

## Data Availability

All the csv files with the experimental data as well as all the Python scripts required for generating the results presented in this work are accessible at the public GitHub repository associated with this work [26]. This code can be easily modified to analyse other cancer types. The data are provided in electronic supplementary material [27].
